# A cross-specific multiplicative binomial recursive model for the analysis of perinatal mortality in a diallel cross among three varieties of Iberian pig

**DOI:** 10.1038/s41598-020-78346-7

**Published:** 2020-12-03

**Authors:** Luis Varona, José Luis Noguera, Joaquim Casellas, Melani Martín de Hijas, Juan Pablo Rosas, Noelia Ibáñez-Escriche

**Affiliations:** 1 Departamento de Anatomía Embriología y Genética Animal, Instituto Agrolimentario de Aragón (IA2), Facultad de Veterinaria, Universidad de Zaragoza, c/ Miguel Servet 177, 50013 Zaragoza, Spain; 2 Genètica i Millora Animal, Institut de Recerca i Tecnologia Agroalimentàries, 25198 Lleida, Spain; 3 Departament de Ciència Animal i dels Aliments, Universitat Autònoma de Barcelona, 08193 Bellaterra, Spain; 4 Programa de Mejora Genética “Castúa”, INGA FOOD S.A. (Nutreco), Avda. A Rúa, 2 – bajo. Edificio San Marcos, 06200 Almendralejo, Spain; 5 Departamento de Ciencia Animal, Universitat Politècnica de València, 46071 Valencia, Spain

**Keywords:** Genetics, Animal breeding

## Abstract

Perinatal piglet mortality is an important factor in pig production from economic and animal welfare perspectives; however, the statistical analysis of mortality is difficult because of its categorical nature. Recent studies have suggested that a binomial model for the survival of each specific piglet with a logit approach is appropriate and that recursive relationships between traits are useful for taking into account non-genetic relationships with other traits. In this study, the recursive binomial model is expanded in two directions: (1) the recursive phenotypic dependence among traits is allowed to vary among groups of individuals or crosses, and (2) the binomial distribution is replaced by the multiplicative binomial distribution to account for over or underdispersion. In this study, five recursive multiplicative binomial models were used to obtain estimates of the Dickerson crossbreeding parameters in a diallel cross among three varieties of Iberian pigs [Entrepelado (EE), Torbiscal (TT), and Retinto (RR)]. Records (10,255) from 2110 sows were distributed as follows: EE (433 records, 100 sows), ER (2336, 527), ET (942, 177), RE (806, 196), RR (870, 175), RT (2450, 488), TE (193, 36), TR (1993, 359), and TT (232, 68). Average litter size [Total Number Born (TNB)] and number of stillborns (SB) were 8.46 ± 2.27 and 0.25 ± 0.72, respectively. The overdispersion was evident with all models. The model with the best fit included a linear recursive relationship between TNB and the logit of $\phi$ of the multiplicative binomial distribution, and it implies that piglet mortality increases with litter size. Estimates of direct effects showed small differences among populations. The analysis of maternal effects indicated that the dams whose mothers were EE had a larger SB, while dams with RR mothers reduced the probability of born dead. The posterior estimates of heterosis suggested a reduction in SB when the sow is crosbred. The multiplicative binomial distribution provides a useful alternative to the binomial distribution when there is overdispersion in the data. Recursive models can be used for modeling non-genetic relationships between traits, even if the phenotypic dependency between traits varies among environments or groups of individuals. Piglet perinatal mortality increased with TNB and is reduced by maternal heterosis.

## Introduction

Perinatal piglet mortality is a major issue in pig production from economic and animal welfare perspectives^1^. The number of stillborn (SB) is a categorical trait that includes a large proportion of zeros. Therefore, the statistical analysis of piglet mortality, as a maternal trait, is complicated because its distribution violates the Gaussian assumption of the standard mixed-model analysis. Varona and Sorensen^2^ calculated the degree of adjustment for several probability distributions (Poisson, Binomial, and Negative Binomial) in their standard version and with zero-inflated approaches. They concluded that a binomial distribution with logit link function to model the survival of each specific piglet within each litter was the most appropriate. However, one of the main limitations of models based on non-Gaussian distributions, such as the binomial, is the difficulty of accounting for non-genetic relationships between traits. Several studies have reported a strong relationship between litter size [Total Number Born (TNB)] and piglet mortality^3–6^, which, in some cases, is non-linear^7–9^. To address the relationship between litter size and piglet mortality, Varona and Sorensen^8^ suggested the use of recursive models^10,11^, because TNB is mainly determined by ovulation rate and embryo losses in early pregnacy^12^, while most stillborns take place around farrowing. Therefore, it is reasonable to postulate a recursive relationship between TNB and SB, under the assumption that mortality around farrowing is phenotypically determined by litter size.

The model proposed by Varona and Sorensen^8^ assumes a general recursive relationship for all data; however, a variety of genetic and non-genetic factors might influence the phenotypic dependence between traits, and it would be interesting to define a model that allows the phenotypic dependence among traits to vary among specific factors. Furthermore, the response variable (i.e., SB) may not strictly follow a binomial distribution, as it may show some degree of over or underdispersion. The binomial distribution can be generalized to the multiplicative binomial (MBN) distribution^13,14^, which can adequately consider over and under-dispersion with an additional parameter ($\theta$).

Therefore, the objective of this study was to develop a recursive model that allows specific recursive functions for a group of individuals or data, and that replaces the binomial distribution with the multiplicative binomial distribution. Specifically, the model was applied to TNB and SB within a full diallel cross^15^ among three varieties of Iberian pig [Entrepelado (EE), Retinto (RR), and Torbiscal (TT)]. The model of analysis had allowed up to nine specific recursive relationships, one for each diallel cross. In addition, the estimates of the recursive parameters were reparametrized to quantify the direct, maternal, and heterosis effects of the Dickerson model^16^ for TNB and SB.

## Methods

### Animals and experimental design

The dataset comprised 10,255 records for TNB and SB from 2110 sows and is a survey derived from the purebred and crossbred populations generated in a full diallel cross carried out by the INGA FOOD S. A. and which included three varieties of Iberian pig [Retinto (RR), Torbiscal (TT), and Entrepelado (EE)] and their reciprocal crosses [Retinto × Torbiscal (RT), Torbiscal × Retinto (TR), Retinto × Entrepelado (RE), Entrepelado × Retinto (ER), Torbiscal × Entrepelado (TE), and Entrepelado × Torbiscal (ET)]. The three varieties are recognized in Spain’s official Iberian herd-book [Asociación Española de Criadores de Ganado Porcino Selecto Ibérico Puro y Tronco Ibérico (AECERIBER)]. A detailed description of their characteristics is provided by Ibáñez-Escriche et al.^17^. SB was recorded by counting piglets born dead in assisted deliveries and with clear indications of stillborn (remains of the placenta, condition of their hooves, general appearance of the piglet) in unattended births. For a detailed description of the experimental protocol of the diallel cross, see Noguera et al.^18^. The sows were in a commercial farm in which purebred and crossbred Iberian sows were mated with boars from a Duroc population following the standard production system of Iberian pigs under intensive management. The distribution of the data among crosses, and the average and standard deviation of TNB and SB for all crosses are presented in Table 1. Besides, the pedigree was extended back up to three generations and included 4609 individuals. The number of founders per population was 47 EE (13 sires and 34 dams), 80 RR (18 sires and 62 dams), and 107 TT (38 sires and 69 dams).

**Table 1:** Phenotypic records of farrowings by Iberian pig breed of sow, and the mean and standard deviation (SD) of the number stillborn (SB) and the total number born (TNB).

EE	100	433	0.22 (0.67)	7.95 (2.34)
ER	527	2336	0.22 (0.67)	8.53 (2.27)
ET	177	942	0.21 (0.87)	8.02 (2.25)
RE	196	806	0.29 (0.88)	8.84 (2.45)
RR	175	870	0.35 (0.88)	8.38 (2.37)
RT	488	2450	0.29 (0.79)	8.60 (2.43)
TE	36	193	0.35 (1.05)	8.69 (2.49)
TR	343	1993	0.24 (0.64)	8.53 (2.32)
TT	68	232	0.38 (0.92)	7.28 (2.31)
TOTAL	2110	10,255	0.25 (0.72)	8.46 (2.27)

### Statistical models

**Figure 1:**
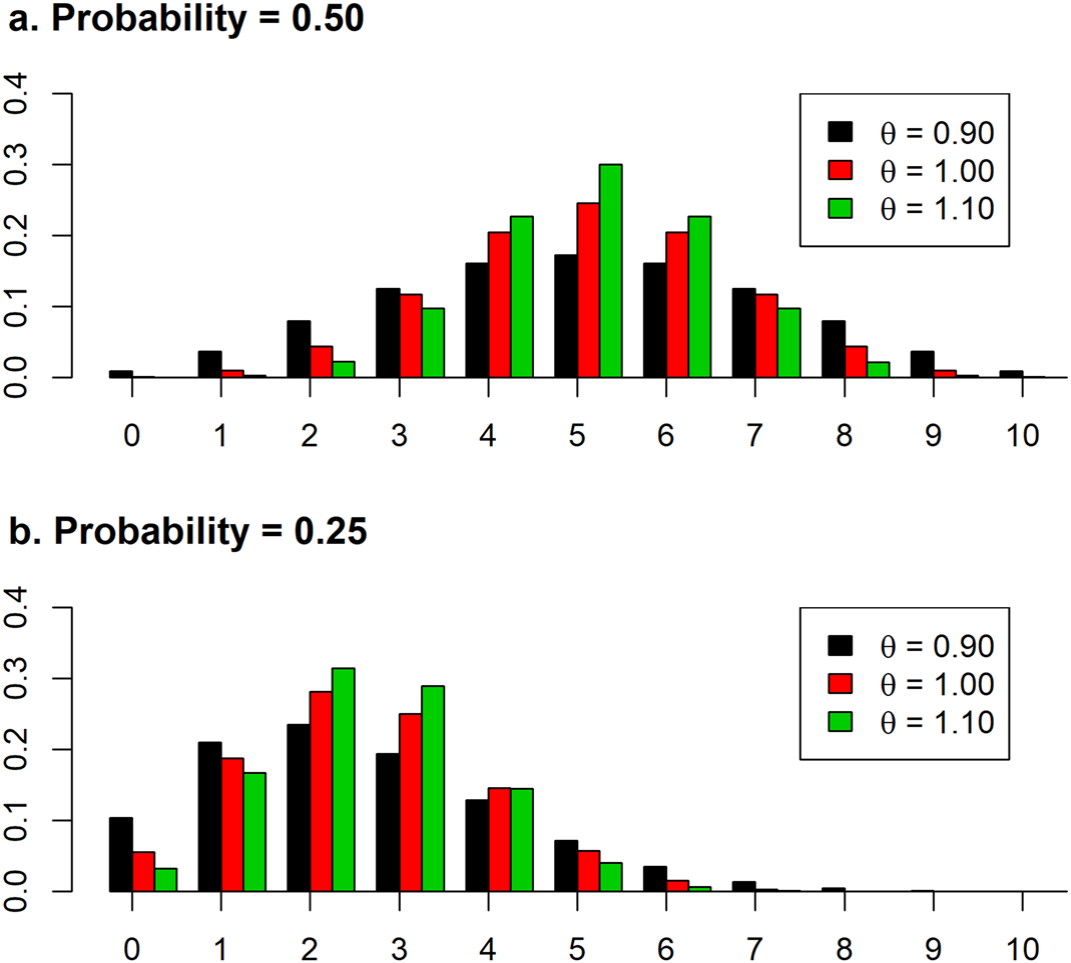
Probability density of the multiplicative binomial distribution for the probability of the Bernoulli events of 0.50 (**a**) and 0.25 (**b**) with values of the θ parameter of 0.90, 1.00, and 1.10.

The multiplicative binomial (MBN) distribution is a generalization of the binomial distribution with two parameters ($\phi \; {\text{and}} \;\theta )$ with the following probability density^13,14^:

$$MBN\left( {x{|}nb,\phi ,\theta } \right) = \frac{{\left( {\begin{array}{*{20}c} nb \\ x \\ \end{array} } \right)\phi^{x} \left( {1 - \phi } \right)^{nb - x} \theta^{{x\left( {nb - x} \right)}} }}{{\mathop \sum \nolimits_{j = 0}^{nb} \left( {\begin{array}{*{20}c} nb \\ j \\ \end{array} } \right)\phi^{j} \left( {1 - \phi } \right)^{nb - j} \theta^{{j\left( {nb - j} \right)}} }},$$

where *x* is the number of positive or negative Bernoulli events (i.e. number of stillborn), *nb* is the number of Bernoulli events, and $\phi \;{\text{and}} \;\theta$ are the parameters of the MBN distribution. The first parameter ($\phi )$ ranged between 0 and 1 as in the binomial distribution, whereas the second ($\theta$) is greater than 0, and it is associated with the degree of over or underdispersion. The MBN distribution is equivalent to the binomial distribution with $\theta$ = 1, and it presents overdispersion when *θ* < 1 and under-dispersion if *θ* > 1. The probability of success of each Bernoulli event with the MBN distribution depends on $\phi \;{\text{and}} \;\theta$ and can be calculated as^19^:

$$p = \phi \frac{{K_{nb - 1} \left( {\phi ,\theta } \right)}}{{K_{nb} \left( {\phi ,\theta } \right)}},$$

with.

$$K_{nb - a} \left( {\phi ,\theta } \right) = \mathop \sum \limits_{j = 0}^{nb - a} \left( {\begin{array}{*{20}c} {nb - a} \\ j \\ \end{array} } \right)\phi^{j} \left( {1 - \phi } \right)^{nb - a - j} \theta^{{\left( {j + a} \right)\left( {nb - a - j} \right)}} ,$$

where *a* is 0 for *K*_*nb*_ and 1 for *K*_*nb−1*_, and *j* is the loop variable in the summation. The effect of different values of the *θ* parameter in the MBN with probabilities of success of each Bernoulli event of 0.25 and 0.5 and with *nb* = 10 is presented in Fig. 1.

In this study, SB (${\varvec{y}} = \left\{ {y_{i} } \right\}$) and TNB (${\varvec{t}} = \left\{ {t_{i} } \right\}$)) were measured at each of the 10,255 litters and they were analyzed under the assumption that the conditional distribution of ***y*** is the following product of multiplicative binomial distributions:

$$f\left( {{\varvec{y}}{|}{\varvec{t}},{\varvec{\phi}},\theta } \right) = \mathop \prod \limits_{i = 1}^{N} MBN\left( {y_{i} {|}t_{i} ,\phi_{i} ,\theta } \right) = \mathop \prod \limits_{i = 1}^{N} \frac{{\left( {\begin{array}{*{20}c} {t_{i} } \\ {y_{i} } \\ \end{array} } \right)\phi_{i}^{{y_{i} }} \left( {1 - \phi_{i} } \right)^{{t_{i} - y_{i} }} \theta^{{y_{i} \left( {t_{i} - y_{i} } \right)}} }}{{\mathop \sum \nolimits_{j = 0}^{{t_{i} }} \phi_{i}^{j} \left( {1 - \phi_{i} } \right)^{{t_{i} - j}} \theta^{{j\left( {t_{i} - j} \right)}} }},$$

where ${\varvec{\phi}} = \left\{ {\phi_{i} } \right\}$ is the vector of the 10,255 parameters of the MBN distribution associated with each litter (*i*), *N* is the number of data, *t*_*i,*_ and *y*_*i*_are the total number of piglets and stillborn in the *i*th litter, respectively. The $\theta$ parameter was assumed to be the same for all litters and represented the general pattern of over or underdispersion.

Under a hierarchical Bayesian framework, a second hierarchy of the model postulated the following linear model for ***t:***

$$\varvec{ t} = {\varvec{Xb}}_{{\varvec{t}}} + {\varvec{Zu}}_{{\varvec{t}}} + {\varvec{Zp}}_{{\varvec{t}}} + {\varvec{e}}_{{\varvec{t}}} ,$$

where ${\varvec{b}}_{{\varvec{t}}}$is the vector of the following systematic effects: type of cross (nine levels: EE, ER, ET, RE, RR, RT, TE, TR, and TT), order of parity (six levels: first, second, third, fourth, fifth, and sixth and beyond), and year-season (YS) (30 levels). In addition, ***u***_***t***_ and ***p***_***t***_ are the vectors of additive genetic (4609 levels) and dam permanent environmental effects (2110 levels), respectively, and ***e***_***t***_ is the vector of gaussian residuals.

In addition, the logit of $\phi$ was modeled linearly in five ways**.** Model I included analogous systematic (***b***_***l***_) and random effects (***u***_***l***_ and ***p***_***l***_**)** that were used in the model for litter size, as follows:

$$\varvec{logit }\phi = {\varvec{Xb}}_{{\varvec{l}}} + {\varvec{Zu}}_{{\varvec{l}}} + {\varvec{Zp}}_{{\varvec{l}}} ;$$

model II incorporated a recursive parameter ($\lambda_{1} )$ with the vector of deviations (***d***) of TNB from the average litter size ($\overline{t}$) across all crosses and order of parities:

$$\varvec{logit }\phi = {\varvec{Xb}}_{{\varvec{l}}} + {\varvec{Zu}}_{{\varvec{l}}} + {\varvec{Zp}}_{{\varvec{l}}} + \lambda_{1} \varvec{d }{;}$$

model III included an additional recursive parameter ($\lambda_{2} )$:

$$\varvec{logit }\phi = {\varvec{Xb}}_{{\varvec{l}}} + {\varvec{Zu}}_{{\varvec{l}}} + {\varvec{Zp}}_{{\varvec{l}}} + \lambda_{1} {\varvec{d}} + \lambda_{2} {\varvec{d}}^{2} ;$$

model IV and model V included cross-specific linear ($\lambda_{1k} )$ and quadratic ($\lambda_{2k} )$ recursive parameters, where *k* has nine levels, which correspond to crosses EE, ER, ET, RE, RR, RT, TE, TR, and TT. Thus, the models were as follows:

$$\begin{aligned} \varvec{logit }\phi & = {\varvec{Xb}}_{{\varvec{l}}} + {\varvec{Zu}}_{{\varvec{l}}} + {\varvec{Zp}}_{{\varvec{l}}} + \mathop \sum \limits_{{{\varvec{k}} = 1}}^{9} \lambda_{1k} {\varvec{w}}_{{\varvec{k}}} \quad \left( {\text{model IV}} \right),\;{\text{ and}} \\ \varvec{logit }\phi & = {\varvec{Xb}}_{{\varvec{l}}} + {\varvec{Zu}}_{{\varvec{l}}} + {\varvec{Zp}}_{{\varvec{l}}} + \mathop \sum \limits_{k = 1}^{9} \lambda_{1k} {\varvec{w}}_{{\varvec{k}}} + \mathop \sum \limits_{k = 1}^{9} \lambda_{2k} {\varvec{w}}_{{\varvec{k}}}^{2} \; \left( {\text{model V}} \right), \\ \end{aligned}$$

the vector ***w***_***k***_ included the deviations of the litter size from the average litter size ($\overline{t})$ if the individual belongs to the *kth* cross, and 0 otherwise.

The prior distributions for additive genetic and permanent environmental effects were assumed as follows:

$$\left( {\begin{array}{*{20}c} {{\varvec{u}}_{{\varvec{l}}} } \\ {{\varvec{u}}_{{\varvec{t}}} } \\ \end{array} } \right)\sim {\varvec{N}}\left( {0,{\varvec{A}} \otimes {\varvec{G}}} \right) \quad \left( {\begin{array}{*{20}c} {{\varvec{p}}_{{\varvec{l}}} } \\ {{\varvec{p}}_{{\varvec{t}}} } \\ \end{array} } \right)\sim {\varvec{N}}\left( {0,{\varvec{I}} \otimes {\varvec{P}}} \right),$$

where **A** is the numerator relationship matrix, and

$${\varvec{G}} = \left( {\begin{array}{*{20}c} {\sigma_{al}^{2} } & {\sigma_{alt} } \\ {\sigma_{alt} } & {\sigma_{at}^{2} } \\ \end{array} } \right)\user2{ }\quad {\varvec{P}} = \left( {\begin{array}{*{20}c} {\sigma_{pl}^{2} } & {\sigma_{plt} } \\ {\sigma_{plt} } & {\sigma_{pt}^{2} } \\ \end{array} } \right),$$

where $\sigma_{al}^{2}$ and $\sigma_{at}^{2}$ are the additive genetic variances for the logit of ${\varvec{\phi}}$ and TNB, and $\sigma_{alt}$ is the covariance between them. Furthermore, $\sigma_{pl}^{2}$ and $\sigma_{pt}^{2}$ are the permanent environmental variances for the logit of ${\varvec{\phi}}$ and TNB, and $\sigma_{plt}$ is the covariance between them. The prior distribution for systematic effects (***b***_***t***_ and ***b***_***l***_), ***G*, *P,*** and the residual variance of litter size ($\sigma_{et}^{2} )$ were assumed uniform within appropriate bounds.

All models were implemented with a Gibbs sampler^20^ with a Metropolis-Hasting step^21^ to sample for the logit and using own software developed in FORTRAN90. The analysis was performed with a single long chain of 550,000 iterations after the first 50,000 were discarded. Convergence was confirmed by visual inspection of the chains and by the CODA package in R software^22^. The Gibbs sampler provides the marginal posterior distributions of all unknowns in the models of analysis that include systematic, additive genetic and permanent environmental effects, as well as variance components and parameters of the MBN distribution.

### Calculation of the Dickerson model parameters

The posterior distributions of the parameters of the Dickerson model were obtained by transforming the output of the cross effects in each iteration of the Gibbs Sampler using a property of the Bayesian analysis that allows to calculate the posterior distribution of any linear transformation of the parameters of the model. Therefore, the direct-line (*D*_*x*_*(E), D*_*x*_*(R), D*_*x*_*(T)*), maternal (*M*_*x*_*(E), M*_*x*_*(R), M*_*x*_*(T)*) for Entrepelado (EE), Retinto (RR) and Torbiscal (TT), and the heterosis for Entrepelado and Retinto (*H*_*x*_*(ER)*), Entrepelado and Torbiscal (*D*_*x*_*(ET)*) and Retinto and Torbiscal (*D*_*x*_*(RT)*) were calculated for TNB (*x* = *t*) and the logit of the $\phi$ parameter of the multiplicative binomial (*x* = *l*). The samples for the Dickerson model parameters at each iteration of the Gibbs sampler were obtained by solving the following system of equations:

$$\left( {\begin{array}{*{20}l} 1 \hfill \quad & 0 \hfill \quad & 0 \hfill \quad & 1 \hfill \quad & 0 \hfill \quad & 0 \hfill \quad & 0 \hfill \quad & 0 \hfill \quad & 0 \hfill \\ {0.5} \hfill \quad & {0.5} \hfill \quad & 0 \hfill \quad & 0 \hfill \quad & 1 \hfill \quad & 0 \hfill \quad & 1 \hfill \quad & 0 \hfill \quad & 0 \hfill \\ {05} \hfill \quad & 0 \hfill \quad & {0.5} \hfill \quad & 0 \hfill \quad & 0 \hfill \quad & 1 \hfill \quad & 0 \hfill \quad & 1 \hfill \quad & 0 \hfill \\ {0.5} \hfill \quad & {0.5} \hfill \quad & 0 \hfill \quad & 1 \hfill \quad & 0 \hfill \quad & 0 \hfill \quad & 1 \hfill \quad & 0 \hfill \quad & 0 \hfill \\ 0 \hfill \quad & 1 \hfill \quad & 0 \hfill \quad & 0 \hfill \quad & 1 \hfill \quad & 0 \hfill \quad & 0 \hfill \quad & 0 \hfill \quad & 0 \hfill \\ 0 \hfill \quad & {05} \hfill \quad & {0.5} \hfill \quad & 0 \hfill \quad & 0 \hfill \quad & 1 \hfill \quad & 0 \hfill \quad & 0 \hfill \quad & 1 \hfill \\ {05} \hfill \quad & 0 \hfill \quad & {0.5} \hfill \quad & 1 \hfill \quad & 0 \hfill \quad & 0 \hfill \quad & 0 \hfill \quad & 1 \hfill \quad & 0 \hfill \\ 0 \hfill \quad & {0.5} \hfill \quad & {0.5} \hfill \quad & 0 \hfill \quad & 1 \hfill \quad & 0 \hfill \quad & 0 \hfill \quad & 0 \hfill \quad & 1 \hfill \\ 0 \hfill \quad & 0 \hfill \quad & 1 \hfill \quad & 0 \hfill \quad & 0 \hfill \quad & 1 \hfill \quad & 0 \hfill \quad & 0 \hfill \quad & 0 \hfill \\ \end{array} } \right)\left( {\begin{array}{*{20}c} {D_{x} \left( E \right)} \\ {D_{x} \left( R \right)} \\ {D_{x} \left( T \right)} \\ {M_{x} \left( E \right)} \\ {M_{x} \left( R \right)} \\ {M_{x} \left( T \right)} \\ {H_{x} \left( {ER} \right)} \\ {H_{x} \left( {ET} \right)} \\ {H_{x} \left( {RT} \right)} \\ \end{array} } \right) = \left( {\begin{array}{*{20}c} {b_{x} \left( {EE} \right)} \\ {b_{x} \left( {ER} \right)} \\ {b_{x} \left( {ET} \right)} \\ {b_{x} \left( {RE} \right)} \\ {b_{x} \left( {RR} \right)} \\ {b_{x} \left( {RT} \right)} \\ {b_{x} \left( {TE} \right)} \\ {b_{x} \left( {TR} \right)} \\ {b_{x} \left( {TT} \right)} \\ \end{array} } \right)$$

where *b*_*x*_*(EE), b*_*x*_*(ER), b*_*x*_*(ET), b*_*x*_*(RE), b*_*x*_*(RR), b*_*x*_*(RT), b*_*x*_*(TE), b*_*x*_*(TR)* and *b*_*x*_*(TT)* were the samples for the nine levels of the systematic effects for litter size (*x* = *t*) or the logit of the $\phi$ parameter of the multiplicative binomial (*x* = *l*).

### Model comparison

The models were compared by the logarithm of the conditional predictive ordinate (LogCPO)^23^. If we consider the vector ${\varvec{y}} = \left( {y_{i} ,{\varvec{y}}_{ - i} } \right)$, where *y*_i_ is the *i*th datum, and ${\varvec{y}}_{ - i}$ is the vector of the data with the *ith* datum deleted, the conditional predictive distribution has the following probability density:

$$p\left( {y_{i} {|}{\varvec{y}}_{ - i} } \right) = \smallint p\left( {y_{i} {|}{\varvec{y}}_{ - i} ,\theta } \right)p\left( {\theta {|}{\varvec{y}}_{ - i} } \right)d\theta ,$$

where $\theta$ is the vector of the unknown parameters in the model. Therefore, $p\left( {y_{i} {|}{\varvec{y}}_{ - i} } \right)$ is the probability of each datum given the rest of the data, which is the conditional predictive ordinate (CPO) for the *i*th datum. The pseudo log-marginal probability of the data is $\mathop \sum \limits_{i} lnp\left( {y_{i} {|}{\varvec{y}}_{ - i} } \right)$.

A Monte Carlo approximation of the CPO suggested by Gelfand^22^ is $\mathop \sum \limits_{i} ln\hat{p}\left( {y_{i} {|}{\varvec{y}}_{ - i} } \right),$ where $\hat{p}\left( {y_{i} {|}{\varvec{y}}_{ - i} } \right) = N\left[ {\mathop \sum \limits_{j = 1}^{Nm} \frac{1}{{p\left( {y_{i} {|}\theta^{j} } \right)}}} \right]^{ - 1}$ , and *Nm* is the number of Markov Chain Monte Carlo (MCMC) draws, and $\theta^{j}$ is the *j*th draw from the posterior distribution of the corresponding parameter. The higher the value of the LogCPO, the better the fit of the model to the data.

### Ethics approval and consent to participate

The research ethics committee of the Institute of Agrifood Research and Technology (IRTA) approved all of the management and experimental procedures involving live animals, which were performed in accordance with the Spanish Policy of Animal Protection RD1201/05, which complies with the European Union Directive 86/609 for the protection of animals used in experimentation.

## Results and discussion

The estimates of the variance components and the differences between the LogCPOs with respect to the best model are presented in Table 2. Comparison of the models indicated that the model with the best fit was model II, which proposes a linear phenotypic dependence between TNB and the logit of *Φ*. Under this model, the slope of the recursive parameter has a posterior mean of 0.257 logit units per piglet with a posterior standard deviation of 0.007. This positive slope implies that the probability of stillborn increases with litter size, confirming the results of previous studies^7,8,24^. It can be caused by a prolonged farrowing, which increases the risk of hypoxia^25^ or influenced by litter weight or average piglet weight^24,26,27^. The differences in LogCPO among the models that included a recursive relationship between TNB and the logit of the $\it \Phi$ parameter of the MBN distribution (models II, III, IV, and V) were small (17.93 LogCPO units). However, there is no evidence of non-linearity (models III and V) or variability of this phenotypic dependence among crosses (models IV and V). The posterior mean estimates of the recursive relationship in more complex models, such as model V (see Supplementary Fig. 1a), may suggest a cross specific non-linear relationship between litter size and the logit of *Φ*. Nevertheless, the differences with the posterior estimate of the general recursive relationship defined in model II (see Supplementary Fig. 1b) were small. Therefore, the logCPO method for model comparison weights the goodness of fit and complexity of the proposed models and it applied Occam’s razor criteria for model comparison and selected a less parameterized model as the best. Furthermore, the ability of the recursive models to capture the non-genetic relationship between traits is illustrated by the large difference in LogCPO (302.92 units) between model II and model I, which considers only additive genetic and permanent environmental covariance between traits.

**Table 2:** Posterior mean (posterior standard deviation) estimates of the variance components and results of the model comparison test (LogCPO) for models I to V.

Model	${\sigma }_{al}^{2}$	${\sigma }_{at}^{2}$	${r}_{a}$	${\sigma }_{pl}^{2}$	${\sigma }_{pt}^{2}$	${r}_{p}$	${\sigma }_{et}^{2}$	*θ*	LogCPO
I	0.107 (0.068)	0.448 (0.143)	0.507 (0.206)	0.753 (0.083)	0.338 (0.096)	0.524 (0.113)	4.502 (0.071)	0.892 (0.008)	− 302.92
II	0.050 (0.044)	0.397 (0.090)	0.203 (0.271)	0.418 (0.056)	0.368 (0.080)	0.028 (0.125)	4.497 (0.071)	0.816 (0.005)	-
III	0.071 (0.038)	0.419 (0.130)	0.223 (0.256)	0.448 (0.056)	0.357 (0.089)	0.022 (0.131)	4.496 (0.071)	0.825 (0.005)	− 0.18
IV	0.038 (0.026)	0.463 (0.162)	0.238 (0.305)	0.448 (0.055)	0.333 (0.103)	0.026 (0.149)	4.495 (0.071)	0.820 (0.008)	− 13.94
V	0.064 (0.035)	0.396 (0.089)	0.226 (0.253)	0.458 (0.055)	0.368 (0.080)	0.031 (0.128)	4.497 (0.070)	0.824 (0.005)	− 17.93

${\sigma }_{al}^{2}$ and ${\sigma }_{pl}^{2} \mathrm{are}$ the additive and permanent environmental variances of the logit of the $\phi$ parameter of the multiplicative binomial distribution and ${\sigma }_{at}^{2}$, ${\sigma }_{pt}^{2}$ and ${\sigma }_{et}^{2}$ are the additive, permanent environmental and residuals variances for Total Number Born (TNB). ${r}_{a}$ and ${r}_{p}$ are the additive genetic and permanent environmental correlations between the logit of the $\phi$ and TNB, *θ* is the parameter of the multiplicative binomial distribution that reflects under or over-dispersion and logCPO is the difference of the logarithm of the conditional predictive ordinate with the best model (model II).

The posterior mean estimates of the *θ* parameter of the MBN distribution ranged between 0.816 (model II) to 0.892 (model I), and, in all models, the posterior distributions were different from 1. Therefore, we found strong evidence of overdispersion that reinforces the adequacy of the MBN distribution rather than the binomial distribution suggested by Varona and Sorensen^8^. The binomial distribution postulates the independence of the series of Bernoulli events that compose the response variable (SB), and this assumption is far from reality in the perinatal mortality of pigs. In this case, the Bernoulli events (survivors or not) are not independent due to a long list of shared biological causes such as, among others, infectious diseases, gestation and farrowing length or stress^24,26^. This phenomenon causes overdispersion with respect to the standard binomial distribution and matches with the postulates argued in the development of the MBN distribution^13,14^, where the values of *θ* < 1 indicate a positive dependence of the events and lead to overdispersion, while values of *θ* > 1 reflect a negative dependence among them and generate under-dispersion with respect the standard binomial distribution.

The results of the variance component estimates were similar for all models (Table 2). For TNB, and with the model with the best fit (model II), the estimates of the posterior mean ± posterior standard deviation of the additive genetic, permanent environmental and residual variance were 0.397 ± 0.090, 0.368 ± 0.080 and 4.497 ± 0.071, respectively. The relative importance of the additive genetic variation was similar to the estimates available from white^6,28,29^ and Iberian^18,30^ pig populations. The estimates of the posterior mean (± posterior standard deviation) for the additive genetic variation of the logit of the $\phi$ parameter was 0.050 ± 0.044, which was substantially less than the posterior mean of the permanent environmental variance (0.418 ± 0.056). The low maternal additive genetic variation in pig mortality was similar to the results of previous studies in white pigs^5,31,32^ and the Iberian population^33^. However, estimates of the additive variances should be taken with caution as the sows come from three different populations. In addition, the posterior estimates of the permanent environmental variances were higher and consistent with the results of Vanderhaeghe et al.^34^, which reported an increase of the risk for stillborn if the sow has had more than one stillborn piglet in a previous farrowing.

The genetic and the permanent environmental correlations between the logit of $\phi$ and TNB were different among the models (Table 2). In model I, the correlations were positive (0.507 and 0.524, respectively) and similar to other estimates^35,36^. The posterior estimates in all of the models that included some type of recursive relationship between TNB and piglet mortality (models II to V) were closer to zero. They ranged from 0.203 to 0.238 for the genetic correlation and from 0.022 to 0.031 for the permanent environmental correlation. However, the Highest Posterior Density at 95% (HPD95) of those estimates always included zero. Those differences among the models were expected because, in model I, the correlation between TNB and logit of $\phi$ can be interpreted directly but, in models II to V, it must be understood as the correlation between TNB and the logit of $\phi$ after the correction for TNB. Furthermore, model I assumed independence between the errors of the two response variables (TNB and SB) and, therefore, the additive and permanent environmental correlations may be higher to compensate for the possible positive environmental correlation between them. In contrast, the remaining models modeled a linear or non-linear dependence between traits and included some degree of residual relationship.

**Figure 2:**
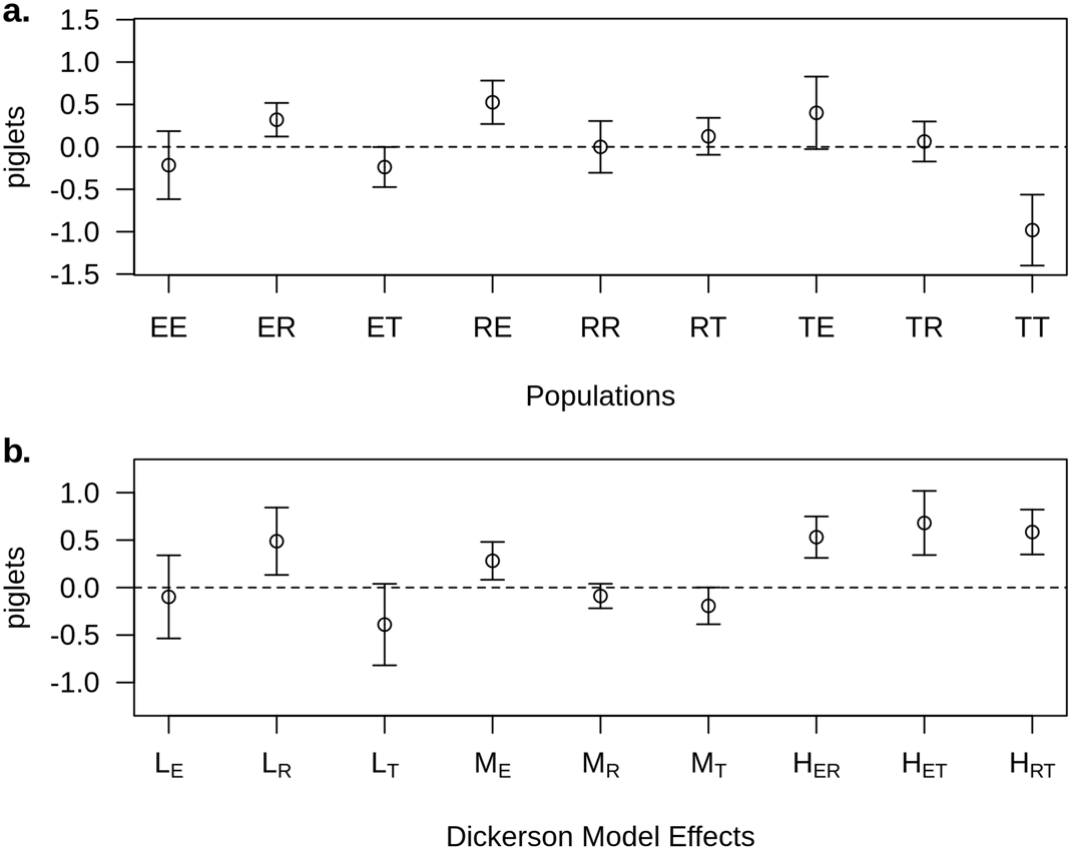
Posterior mean (and Highest Posterior Density at 95%) estimates of the deviation of the cross effects (EE—Entrepelado, ER—Entrepelado-Retinto-, ET—Entrepelado-Torbiscal-, RE—Retinto-Entrepelado-, RR—Retinto, RT—Retinto-Torbiscal-, TE—Torbiscal-Entrepelado-, TR—Torbiscal-Retinto and TT—Torbiscal) with respect to their average (**a**) and of the direct line (L_E_, L_R,_ and L_T_), maternal (M_E_, M_R_ and M_T_), and heterosis (H_ER_, H_ET_, H_RT_) effects of the Dickerson model (**b**) for total number born (TNB) under model II.

**Figure 3:**
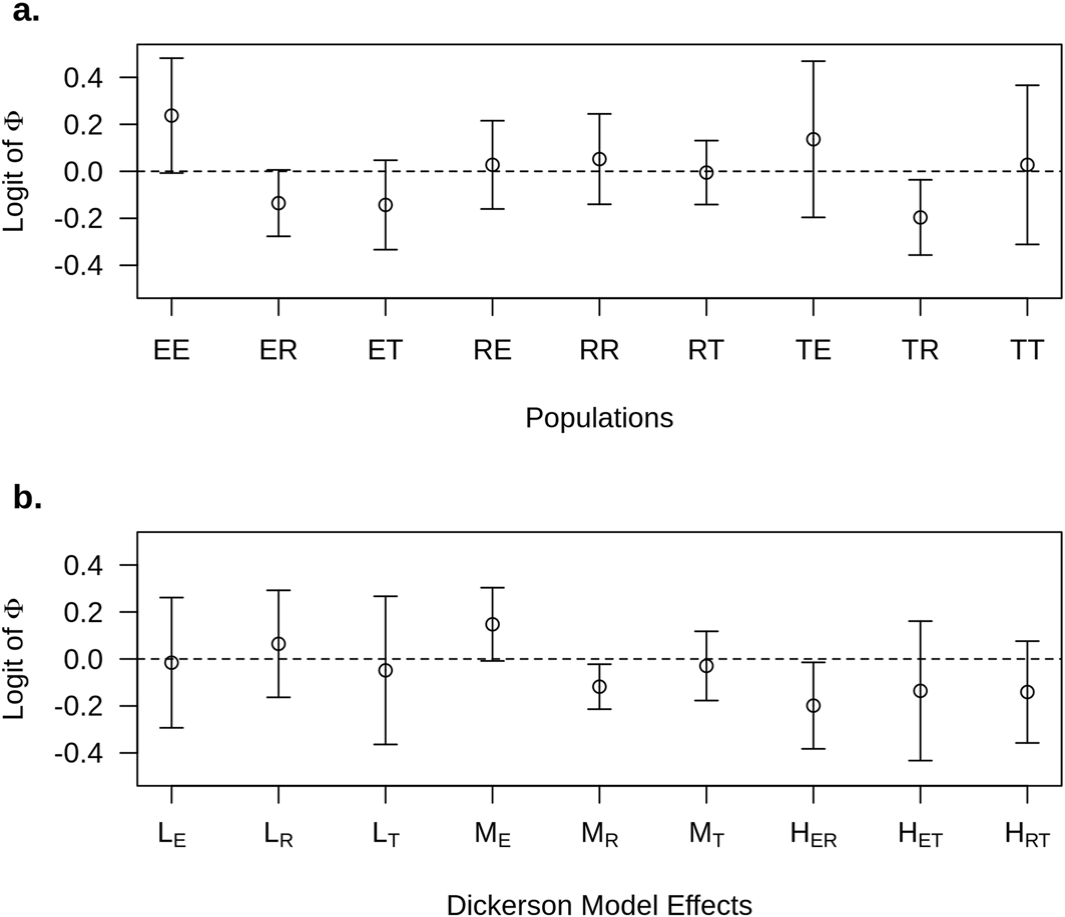
Posterior mean (and highest posterior density at 95%) estimates of the deviation of the cross effects (EE—Entrepelado, ER—Entrepelado-Retinto-, ET—Entrepelado-Torbiscal-, RE—Retinto-Entrepelado-, RR—Retinto, RT—Retinto-Torbiscal-, TE—Torbiscal-Entrepelado-, TR—Torbiscal-Retinto and TT—Torbiscal) with respect to their average (**a**) and of the direct line (L_E_, L_R,_ and L_T_), maternal (M_E_, M_R_ and M_T_), and heterosis (H_ER_, H_ET_, H_RT_) effects of the Dickerson model (**b**) for the logit of the *Φ* parameter of the multiplicative binomial distribution under model II.

The posterior estimates of systematic cross effects under the model that had the best fit (model II) are presented in Fig. 2 and 3. Figure 2a shows the posterior estimates for TNB. The RE and TE crosses were the best and had estimates of the posterior mean ± posterior standard deviation of 0.526 ± 0.128 and 0.401 ± 0.214 piglets above the average, respectively (Fig. 2a). TT was the worst cross and had an estimate of the posterior mean (± posterior standard deviation) of − 0.982 ± 0.209 below the average line effect. Those estimates of cross effects can be re-parameterized in terms of the Dickerson model in the direct, maternal, and heterosis effects (Fig. 2b). The posterior mean estimates of the direct effects ranged from − 0.391 (Torbiscal) to 0.492 (Retinto); the maternal effects ranged from − 0.193 (Torbiscal) to 0.282 (Entrepelado). The posterior mean estimates (± posterior standard deviation) of the effects of heterosis were 0.532 ± 0.109, 0.681 ± 0.169, and 0.584 ± 0.118 between Entrepelado and Retinto, Entrepelado and Torbiscal, and Retinto and Torbiscal, respectively. As expected, the results were similar to those of Noguera et al.^18^, who applied a mixed linear model to the same populations.

Figure 3a shows the posterior mean and standard deviation estimates of the line effects for the logit of *Φ*. It should be noted that altought model II proposes the same slope for all crosses, the procedure was able to detect differences among crosses at the intercept, which was set at the average TNB. The cross with the lowest posterior mean ± posterior standard deviation was the TR with − 0.196 ± 0.080, followed by ET and ER with − 0.143 ± 0.095 and − 0.135 ± 0.709, respectively. The EE and TE crosses had the highest posterior mean ± posterior standard deviation with 0.237 ± 0.122 and 0.136 ± 0.166. Those estimates were re-parameterized in terms of the Dickerson model in the direct, maternal, and heterosis effects (Fig. 3b). Estimates of direct effects ranged between − 0.051 ± 0.159 (Torbiscal) to 0.069 ± 0.112. The results of the maternal effects suggest that the dams that had an Entrepelado mother had more stillborn offspring (0.150 ± 0.077). In contrast, the dams that had a Retinto mother showed an effect in the opposite direction (− 0.120 ± 0.047).

The posterior estimates of the heterosis effects for piglet mortality were always negative and indicates a positive effect on heterosis (a reduction of the probability of stillborn), as expected from previous studies^37^. They ranged between − 0.196 ± 0.091 (Entrepelado and Retinto) to − 0.130 ± 0.147 (Entrepelado and Torbiscal), and the posterior probabilities of a negative heterosis effect were 98.34, 81.05 and 90.14% for Entrepelado and Retinto, Entrepelado and Torbiscal and Retinto and Torbiscal, respectively.

The heterosis in piglet survival observed in our study reinforces the suggestion of Noguera et al.^18^, who recommended using a pyramidal scheme in the production of crossbred Iberian sows. Previous studies^30,38^ have reported the presence of heterosis for litter size in the Iberian pig populations and, our study has shown that crossbred Iberian sows also may provide a lower chance of stillborns. Prolificacy is lower in the Iberian pig populations than it is in the white pig populations^39^. However, efforts to improve the reproductive efficiency of the former are possible given the estimates of genetic variation in the population^18,29^. The success of selection for litter size, however, is limited by maternal ability to rear the extra piglets^40^. Therefore, the exploitation of heterosis for piglet survival can play an important role in ensuring an acceptable survival rate in sows that produce large litters in Iberian pig production.

Finally, it is worth noting that recursive methods are useful means of overcoming one of the main limitations of the use of non-Gaussian distributions, i.e., the difficulty in modeling non-genetic relationships between traits^8^. However, the phenotypic relationship between traits might differ among environments or groups of individuals. In this study, we extended the approach of Varona and Sorensen^8^ with two important novelties: (a) by including several recursive functions that, in this study, were assigned to nine diallel crosses, although the recursive relationships might be defined as nested within each systematic effect in the model, and (b) by replacing the binomial distribution by a multiplicative binomial distribution that is able to consider for the overdispersion. Nevertheless, the results of the statistical analysis did not show that the variability in the recursive relationships was relevant, although we found strong evidence of overdispersion.

The proposed approach has been developed under a hierarchical Bayesian scheme that included: (1) the multiplicative binomial probability of SB given TNB and the parameters of the MBN distribution, (2) The conditional distributions of the logit of the $\phi$ parameters of the MBN distribution and TNB given the additive genetic and systematic effects, the recursive parameters and the residual variance for TNB, (3) The prior distribution of the additive genetic and systematic effects given the additive and permanent environmental variance components, and (4) the prior distributions of the variance components and of the $\theta$ parameter of the MBN distribution. Nevertheless, alternative approaches could be developed by the implementation of a likelihood-based inference using a maximization algorithm such as Expectation–Maximization^41^.

Finally, it is worth to mention that the model used in this study still had several assumptions that should be considered. First, it was assumed that the systematic effects associated with the diallel crosses (EE, ER, ET, RE, RR, RT, TE, TR, and TT) were independent a-priori, although they were related as the sows presents at each cross were genetically linked. Therefore, the additive genetic effects and the cross effect may be somewhat confused. However, we decided to include the additive genetic effects in the models in order to correct for the covariances between individuals within and between crosses. Theoretically, inference for cross effect or Dickerson model parameters must be made from unrelated observations between crosses, that it was not feasible with the available information from the survey. Nevertheless, the genetic relationships among the sows between crosses should have a minimal influence on the results as the additive variance for TNB and SB was very low. Secondly, only linear and quadratic relationships between TNB and the logit of *Φ* were allowed, and, in the literature, some interesting alternative proposals used change-point techniques^42^ to model non-linear recursive relationships^7,43^ that may be used in future studies.

## Conclusions

The multiplicative binomial distribution provides a useful alternative to the binomial distribution when there is overdispersion in the data. Recursive models can be applied for modeling non-genetic relationships between traits across environments or groups of individuals. In the Iberian pig populations, the perinatal piglet mortality measured as the SB increased with TNB and the effects of maternal heterosis on piglet survival are also revealed.

## Supplementary information

Supplementary Information.

## Data Availability

The software and dataset used during the current study are available from the corresponding author on reasonable request.

## Abbreviations

EE: Entrepelado
RR: Retinto
TT: Torbiscal
EE: Entrepelado × Entrepelado
ER: Entrepelado × Retinto
ET: Entrepelado × Torbiscal
RE: Retinto × Entrepelado
RR: Retinto × Retinto
RT: Retinto × Torbiscal
TE: Torbiscal × Entrepelado
TR: Torbiscal × Retinto
TT: Torbiscal × Torbiscal
TNB: Total number born
SB: Stillborn
CPO: Conditional predictive ordinate
HPD95: Highest posterior density at 95%
MBN: Multiplicative binomial distribution
HYS: Herd-year-season
