# Supplementary material for: A cross-specific multiplicative binomial recursive model for the analysis of perinatal mortality in a diallel cross among three varieties of Iberian pig
Source: Sci Rep. 2020 Dec 3;10:21190. doi: 10.1038/s41598-020-78346-7 (PMC7712833; doi:10.1038/s41598-020-78346-7)

**A cross-specific multiplicative binomial recursive model for the analysis of perinatal mortality in a diallel cross among three varieties of Iberian pig.**

Luis Varona^1^, José Luis Noguera^2^, Joaquim Casellas^3^, Melani Martín de Hijas^3^, Juan Pablo Rosas^4^, Noelia Ibáñez-Escriche^5^

*^1^Departamento de Anatomía Embriología y Genética Animal, Instituto Agrolimentario de Aragón (IA2). Universidad de Zaragoza, 50013 Zaragoza, Spain*

*^2^Genètica i Millora Animal, Institut de Recerca i Tecnologia Agroalimentàries, 25198 Lleida, Spain*

*^3^Departament de Ciència Animal i dels Aliments, Universitat Autònoma de Barcelona, 08193 Bellaterra, Spain*

*^4^Programa de Mejora Genética “Castúa”. INGA FOOD S.A. (Nutreco). Avda. A Rúa, 2 – bajo. Edificio San Marcos. 06200 Almendralejo, Spain*

*^5^Departamento de Ciencia Animal, Universitat Politècnica de València, 46071 València, Spain*

**Supplementary Figure 1**

Posterior mean estimate of the recursive relationship between litter size and the logit of the $\phi$ parameter of the multiplicative binomial distribution in models V (1.a) and II (1.b).


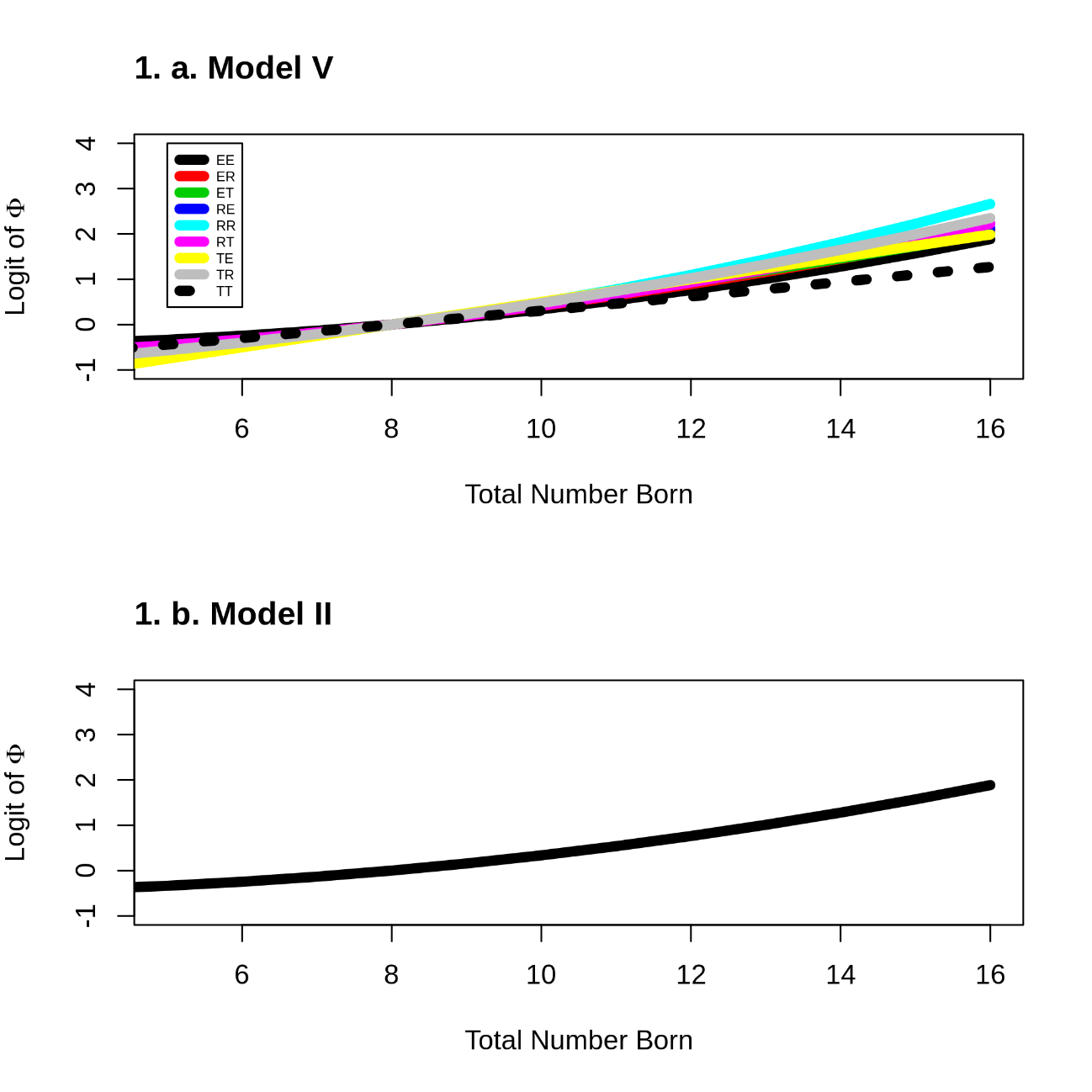

Supplement: Supplementary file 1 — Supplementary Information. [file 41598_2020_78346_MOESM1_ESM.docx]
